# Performance of Symptom-Based Case Definitions to Identify Influenza Virus Infection Among Pregnant Women in Middle-Income Countries: Findings From the Pregnancy and Influenza Multinational Epidemiologic (PRIME) Study

**DOI:** 10.1093/cid/ciaa1697

**Published:** 2021-12-06

**Authors:** Meredith G. Wesley, Yeny Tinoco, Archana Patel, Piyarat Suntarratiwong, Danielle Hunt, Chalinthorn Sinthuwattanawibool, Giselle Soto, Wanitchaya Kittikraisak, Prabir Kumar Das, Carmen Sofia Arriola, Danielle Hombroek, Joshua Mott, Kunal Kurhe, Savita Bhargav, Amber Prakash, Richard Florian, Oswaldo Gonzales, Santiago Cabrera, Edwin Llajaruna, Tana Brummer, Parker Malek, Siddhartha Saha, Shikha Garg, Eduardo Azziz-Baumgartner, Mark G. Thompson, Fatimah S. Dawood

**Affiliations:** 1 Influenza Division, Centers for Disease Control and Prevention, Atlanta, Georgia, USA; 2 US Naval Medical Research Unit No. 6, Bellavista, Peru; 3 Lata Medical Research Foundation, Nagpur, India; 4 Datta Meghe Institute of Medical Sciences, Sawangi, India; 5 Queen Sirikit National Institute of Child Health, Thailand Ministry of Public Health, Bangkok, Thailand; 6 Abt Associates, Atlanta, Georgia, USA; 7 Thailand Ministry of Public Health—US Centers for Disease Control and Prevention Collaboration, Nonthaburi, Thailand; 8 Hospital Nacional Arzobispo Loayza, Lima, Peru; 9 Instituto Nacional Materno Perinatal, Lima, Peru; 10 Hospital Nacional Docente Madre Niño San Bartolomé, Lima, Peru; 11 Hospital Nacional Dos de Mayo, Lima, Peru

**Keywords:** influenza, pregnancy, global health

## Abstract

**Background.:**

The World Health Organization (WHO) recommends case definitions for influenza surveillance that are also used in public health research, although their performance has not been assessed in many risk groups, including pregnant women in whom influenza may manifest differently. We evaluated the performance of symptom-based definitions to detect influenza in a cohort of pregnant women in India, Peru, and Thailand.

**Methods.:**

In 2017 and 2018, we contacted 11 277 pregnant women twice weekly during the influenza season to identify illnesses with new or worsened cough, runny nose, sore throat, difficulty breathing, or myalgia and collected data on other symptoms and nasal swabs for influenza real-time reverse transcription–polymerase chain reaction (rRT-PCR) testing. We calculated sensitivity, specificity, positive-predictive value, and negative-predictive value of each symptom predictor, WHO respiratory illness case definitions, and a de novo definition derived from results of multivariable modeling.

**Results.:**

Of 5444 eligible illness episodes among 3965 participants, 310 (6%) were positive for influenza. In a multivariable model, measured fever ≥38°C (adjusted odds ratio [95% confidence interval], 4.6 [3.1–6.8]), myalgia (3.0 [2.2–4.0]), cough (2.7 [1.9–3.9]), and chills (1.6 [1.1–2.4]) were independently associated with influenza illness. A definition based on these 4 (measured fever, cough, chills, or myalgia) was 95% sensitive and 27% specific. The WHO influenza-like illness (ILI) definition was 16% sensitive and 98% specific.

**Conclusions.:**

The current WHO ILI case definition was highly specific but had low sensitivity. The intended use of case definitions should be considered when evaluating the tradeoff between sensitivity and specificity.

Pregnancy is characterized by unique physiologic changes that may influence the clinical manifestation of influenza virus infections. For example, pregnant women may experience new or increased rhinorrhea [1–3] or increased shortness of breath due to compression of the lungs by a growing uterus [4]. Immunologic changes that occur during pregnancy may also affect clinical manifestation and severity of viral infections including influenza [5]. Pregnant women are thought to be at increased risk for increased complications from influenza and have been identified as a priority group for influenza research [5–7].

Prior studies of seasonal and pandemic influenza during pregnancy have aimed to estimate disease burden, describe clinical features of illness, and evaluate the efficacy of influenza vaccines [8–16] but have used a variety of case definitions, making it challenging to compare results or interpret differences in study findings [8, 9, 16, 17]. Additionally, the relative importance of screening test characteristics such as sensitivity or specificity varies based on the intended use of the definition. For example, studies of disease burden benefit from a case definition with high sensitivity where the likelihood of capturing all true influenza cases is high, while influenza surveillance systems may benefit from a specific case definition to identify circulating virus strains and monitor trends.

Standard case definitions for the detection of influenza may perform differently in pregnant women than in the general population considering the physiological changes that occur during pregnancy. Studies evaluating clinical criteria for detection of influenza are currently limited to nonpregnant adults and children in the community setting [18–20], ambulatory care settings [21–24], or more frequently, the hospitalized setting [25–27]. No study to date has evaluated case definitions for influenza among pregnant women.

During 2017 and 2018, a large, multisite, prospective cohort study was conducted in India, Peru, and Thailand to estimate the incidence of real-time reverse transcription–polymerase chain reaction (rRT-PCR)–confirmed influenza during pregnancy and evaluate its impact on pregnancy outcomes. The study used a broad definition for influenza-like symptoms (ILSs) that included both respiratory and constitutional symptoms and did not require fever. Using data from this study, we evaluated the performance of individual symptoms and symptom combinations as criteria to identify episodes of rRT-PCR–confirmed influenza virus infection among pregnant women in middle-income countries.

## METHODS

### Study Design and Methods

This study uses data from the Pregnancy and Influenza Multinational Epidemiologic (PRIME) Study conducted in Nagpur, India; Lima, Peru; and Bangkok, Thailand. The PRIME study design and methods have been described previously [28].

### Active Surveillance and Illness Reporting Methods

Women were enrolled during pregnancy and followed with active surveillance for ILSs through the end of their pregnancies. An ILS was defined as new onset or sudden worsening of 1 or more of the following symptoms: myalgia, cough, runny nose or nasal congestion, sore throat, or difficulty breathing. At study enrollment, women were given digital thermometers, taught to measure an oral temperature, and asked to measure their temperature daily prior to antipyretic use if they experienced ILSs and record their temperature on a symptom diary card. As part of surveillance, women were contacted twice weekly by phone call or home visits by study staff. At each contact, participants were asked if they had an ILS during the preceding 7 days or since the last surveillance contact if it was more than 7 days before the current contact. Participants were also instructed to contact staff if they experienced ILSs between surveillance contacts.

Once a qualifying illness was identified, an interview was conducted to collect additional information about the illness episode, including the complete constellation of ILSs present and the date of illness onset. If symptom onset occurred within the last 7 days, participants were asked to contribute a midturbinate nasal swab, collected by trained study staff in Peru and India and participant self-collected in Thailand [29].

At the end of the illness or 14 days after illness onset, whichever occurred first, participants were asked to provide follow-up information about the illness episode including development of additional ILSs, presence of measured fever, subjective fever or chills, and highest measured oral temperature if measured fever was reported. Participants were eligible to report a new ILS episode and contribute another nasal swab 14 days or more after the onset of a previously reported ILS episode.

Nasal swab specimens were tested for influenza viruses at local laboratories by rRT-PCR. All study laboratories passed US Centers for Disease Control and Prevention (CDC) proficiency tests and used CDC protocols, primers, and probes.

### Exclusion Criteria

For this analysis, illness episodes were restricted to those that occurred during the influenza season at each site defined as starting and ending on the symptom onset date for the first and last rRT-PCR–confirmed influenza episode at each site during each enrollment year. The analysis only included illness episodes with complete acute illness and follow-up information and a nasal swab collected within 7 days of symptom onset.

### Identification of Case Definitions of Interest

To identify case definitions for evaluation, we used 3 approaches: (1) identification of World Health Organization (WHO)–recommended case definitions, (2) modification of WHO-recommended case definitions to improve 1 or more attributes of case definition performance (eg, sensitivity or specificity of the case definition), and (3) generalized estimating equation (GEE) modeling to identify symptom predictors of influenza within the study cohort that were then used to develop de novo case definitions for exploratory purposes.

We identified 2 WHO-recommended case definitions that have been frequently used to identify influenza in prior research studies and surveillance efforts: influenza-like illness (ILI), defined as measured fever of at least 38.0°C and cough with onset during the prior 10 days, and acute respiratory infection (ARI), defined as new or worsened cough, sore throat, runny nose, or difficulty breathing with onset during the prior 10 days [30, 31]. Prior studies have shown that the WHO ILI case definition has low sensitivity for influenza because it requires a measured fever greater than or equal to 38.0°C, which may be absent with influenza or masked by antipyretic use [27]. Therefore, we studied modified versions of this WHO case definition by expanding the symptom criteria for fever to include (1) subjective or measured fever plus new or worsened cough and (2) subjective or measured fever or chills plus new or worsened cough. Prior studies have also shown that the WHO ARI case definition has low specificity for influenza in both hospitalized patients and community cohorts because of its broad symptom criteria [27, 32]. Therefore, we also evaluated a modified version of the WHO-recommended ARI case definition that required the presence of at least 2 ARI symptoms, hypothesizing this might increase specificity.

To identify de novo case definitions for evaluation, we conducted GEE modeling to determine symptoms as predictors for rRT-PCR–confirmed influenza virus infection among women in the cohort. The GEE models were used to account for participants’ contribution of multiple illness episodes. We compared frequencies of individual symptoms between ILS episodes that tested positive and negative for rRT-PCR–confirmed influenza using chi-square or Fisher’s exact tests. Using statistically significant results of the univariate assessment (ɑ = .05), we developed a multivariable, GEE model using forward stepwise selection controlling for country of site (India, Peru, Thailand), participant age at enrollment in years, educational level (no formal education, primary, secondary, postsecondary/university), influenza vaccination status during the study season, presence of chronic conditions during the prior 24 months, and time in days from illness onset to swab collection. We included interaction terms for effect modification of vaccination status and educational level by country. If collinearity was detected between symptoms, the symptom with the stronger univariate association was selected for the multivariable model. We used the results from this model to guide selection of individual symptoms and symptom combinations as case definitions for evaluation.

### Testing Performance of Case Definitions

For each case definition of interest, we calculated sensitivity, specificity, negative-predictive value (NPV) and positive-predictive value (PPV) with 95% confidence intervals (CIs) among illness episodes since disease prevalence did not vary widely between years or sites (range, 2–5%). rRT-PCR confirmation of influenza was used as the gold standard for all case-definition performance calculations.

## RESULTS

### Illness Episode Characteristics

**Figure 1.**
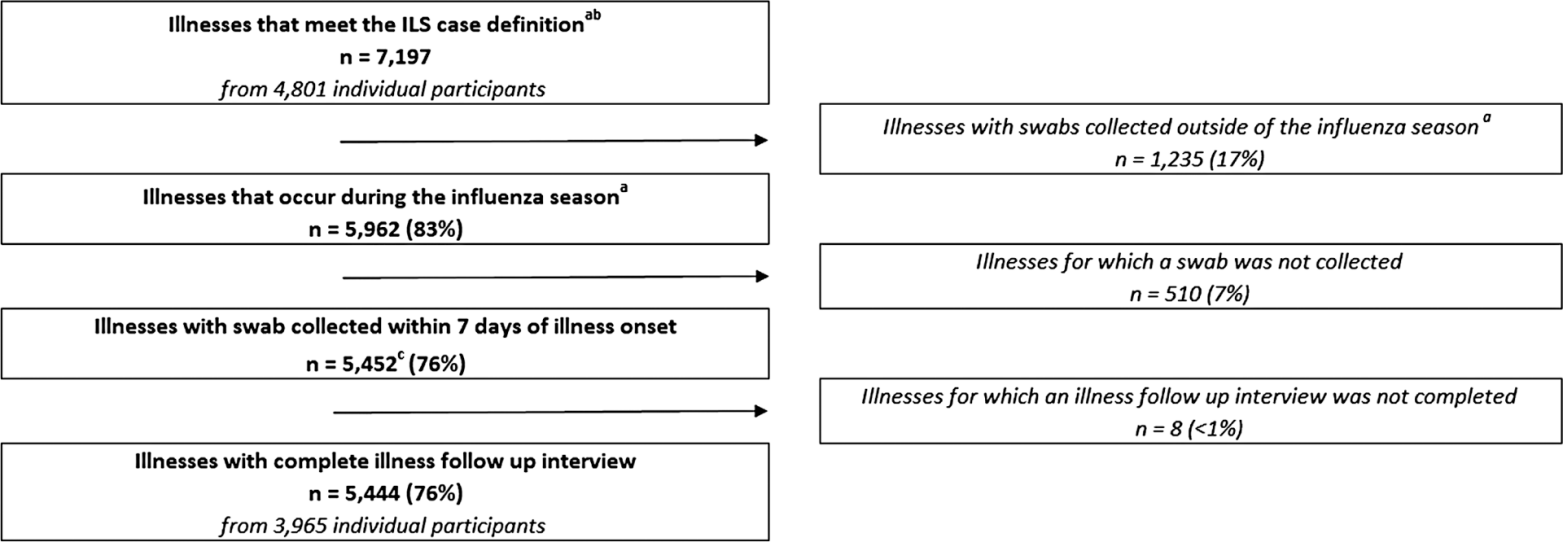
Flow diagram of reported respiratory illnesses, PRIME cohort study, 2017–2018. ^a^Flu season defined as starting and ending on the first day of symptom onset for the first and last rRT-PCR–confirmed influenza illnesses. Influenza seasons defined as: India, year 1: 29 June–27 November 2017; year 2: 14 July–18 November 2018. Peru, year 1: 20 May–22 December 2017; year 2: 22 March–31 December 2018. Thailand, year 1: 22 June 2017–18 January 2018; year 2: 8 June–4 October 2018. ^b^The ILS case definition includes report of new or worsened cough, sore throat, myalgia, runny nose, or difficulty breathing. ^c^One illness excluded for incomplete swab collection information. Abbreviations: ILS, influenza-like symptoms; PRIME, Pregnancy and Influenza Multinational Epidemiologic; rRT-PCR, real-time reverse transcription–polymerase chain reaction.

During 2017 and 2018, the PRIME study enrolled 11 277 pregnant women, of whom 4801 (43%) had 1 or more ILS episodes, resulting in 7197 reported illness episodes. Of these illness episodes, 5444 (76%) were eligible for inclusion in this analysis, including 310 (6%) that were positive for influenza by rRT-PCR (Figure 1). Of all rRT-PCR–confirmed influenza cases, 88% were caused by influenza A viruses (54% influenza A[H1N1] pdm09, 31% A[H3N2] and 3% A [unsubtypable]) and 12% by influenza B (10% B/Yamagata, 2% B/Victoria, and 1% B/unable to lineage type) (Supplementary Table 1).

The 5444 eligible illness episodes included in this analysis were contributed by 3965 unique participants. Compared with participants without rRT-PCR–confirmed influenza, those with influenza were more likely to be from the India or Peru sites, to be older in age, to have higher parity, and to be unvaccinated against influenza during the study season (Table 1). Participants with rRT-PCR–confirmed influenza had more independent ILS episodes for which a swab was contributed, on average, than those without rRT-PCR–confirmed influenza (1.5 swabs vs 1.4 swabs, *P* < .01). Among participants with rRT-PCR–confirmed influenza, 34% experienced 2 or more ILS episodes for which they contributed a nasal swab compared with 27% among participants with no rRT-PCR–confirmed influenza illness (*P* < .01). Illness episodes that tested positive for influenza by rRT-PCR had a shorter mean duration between symptom onset and respiratory specimen collection than those that tested negative (2.2 days vs 2.6 days, *P* < .01).

Among all illness episodes, the most commonly reported symptoms were runny nose (4869/5444, 89%) and sore throat (4174/5444, 77%), whereas the least commonly reported symptom was measured fever ≥38.0°C (186/5444, 3%); the frequency of symptoms varied by country (Supplementary Table 2). In univariate analysis, rRT-PCR–confirmed influenza was associated with subjective fever, measured fever (≥38.0°C), chills, myalgia, new or worsened cough, and difficulty breathing. After controlling for site, age, educational level, current influenza vaccination status, presence of chronic conditions, and days from illness onset to swab collection and interactions between country and education and country and vaccination, measured fever ≥38.0°C (adjusted odds ratio [aOR], 4.6; 95% CI, 3.1–6.8), myalgia (aOR, 3.0; 95% CI, 2.2–4.0), cough (aOR, 2.7; 95% CI, 1.9–3.9), and chills (aOR, 1.6; 95% CI, 1.1–2.4) were significantly associated with rRT-PCR–confirmed influenza (Table 2). Subjective fever was not included in the multivariable model due to collinearity with measured fever, which had a stronger association with influenza positivity by rRT-PCR.

### Case-Definition Performance

**Figure 2.**
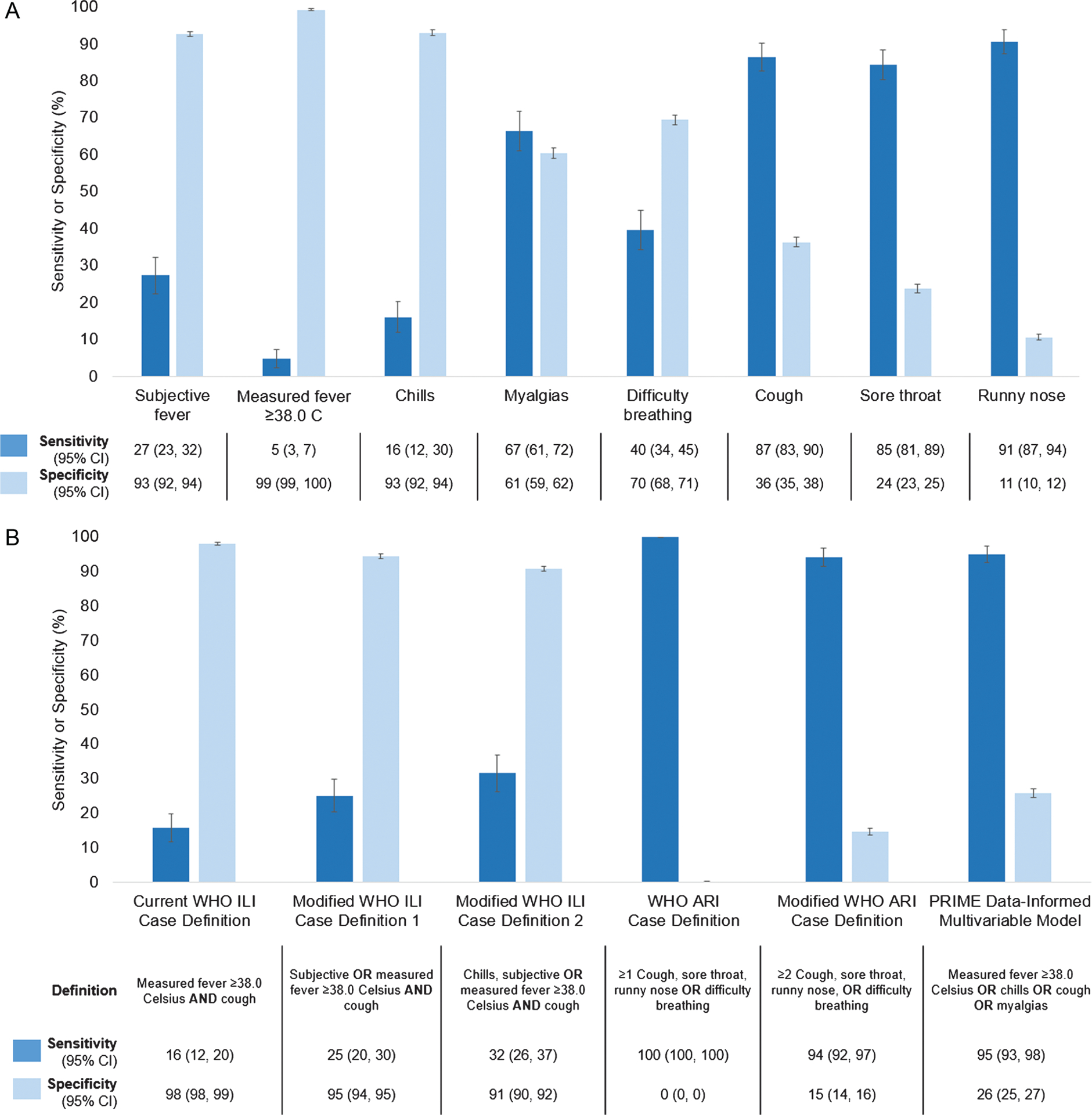
Performance of individual symptoms (*A*) and symptom combination case definitions (*B*) for detection of RT-PCR–confirmed influenza virus infection; sensitivity and specificity; PRIME cohort study, 2017–2018. N = 5444 illness episodes. Abbreviations: ARI, acute respiratory infection; CI, confidence interval; ILI, influenza-like illness; PRIME, Pregnancy and Influenza Multinational Epidemiologic; RT-PCR, reverse transcription–polymerase chain reaction; WHO, World Health Organization.

Sensitivities and specificities of individual symptoms and symptom combination case definitions are shown in Figure 2. Individual constitutional symptoms including measured fever ≥38.0°C, subjective fever, and chills had low sensitivity (range, 5–27%) and high specificity (range, 93–99%). Respiratory symptoms except for difficulty breathing had high sensitivity (range, 85–91%) and varying specificity (Figure 2A). Overall, new or worsened cough had high sensitivity (87%; 95% CI, 83–90%) with moderate specificity (36%; 95% CI, 35–38%). Among all symptoms, runny nose had the highest sensitivity (91%; 95% CI, 87–94%) but the lowest specificity (11%; 95% CI, 10–12%), whereas measured fever had the highest specificity (99%; 95% CI, 99–100%) but the lowest sensitivity (5%; 95% CI, 3–7%).

The current WHO ILI case definition had relatively low sensitivity (16%; 95% CI, 12–20%) but high specificity for rRT-PCR–confirmed influenza (98%; 95% CI, 98–99%) (Figure 2B). Expanding the WHO ILI case definition to include subjective fever resulted in a modest increase in sensitivity (25%; 95% CI, 20–30%) and a reduction in specificity (95%; 95% CI, 94–95%), as did further expansion to include subjective fever or chills (sensitivity, 32%; 95% CI, 26–37%; specificity, 91%; 95% CI, 90–92%). In contrast, the WHO ARI case definition was 100% sensitive and had almost no specificity since the study case definition included all the ARI case-definition symptoms plus myalgia and there were no episodes of rRT-PCR–confirmed influenza in which participants had myalgia alone. Modifying the WHO ARI case definition to require at least 2 symptoms resulted in a slight decrease in sensitivity (94%; 95% CI, 92–97%) and increase in specificity (15%; 95% CI, 14–16%). The de novo case definition developed from the GEE modeling and defined as at least 1 of measured fever (≥38°C), chills, cough, or myalgia had high sensitivity (95%; 95% CI, 93–98%) and moderate specificity (26%; 95% CI, 25–27%) (Figure 2B). As a sensitivity analysis, this definition was simplified to measured fever (≥38.0°C) or cough, which were the systemic and respiratory symptoms with the strongest association with influenza, respectively, and sensitivity was reduced (88%; 95% CI, 85–92%) and specificity was increased moderately (36%; 95% CI, 37–39%) (data not shown).

The prevalence of rRT-PCR–confirmed influenza at study sites in 3 countries was consistently between 2% and 5% across study years and seasons, so differences by site or year in PPV and NPV by disease prevalence could not be observed. Overall, individual symptoms performed with relatively low PPV (range, 6–30%) and relatively high NPV (range, 95–98%) as did combination symptom case definitions (PPV range, 6–35%; NPV range, 95–100%) (Table 3). The WHO ILI case definition performed with the highest PPV (35%; 95% CI, 27–43%) and high NPV (95%; 95% CI, 95–96%).

## DISCUSSION

To date, no assessment of case definitions for detection of influenza among pregnant women has been published. This study addresses this gap using data from more than 5000 respiratory illness episodes among pregnant women during local influenza seasons to evaluate existing and modified versions of the WHO ILI and ARI case definitions and several alternative single and multisymptom case definitions for the detection of rRT-PCR– confirmed influenza.

The current WHO ILI case definition was highly specific for the detection of rRT-PCR–confirmed influenza but had low sensitivity even after expanding the definition of fever to include subjective fever or chills in addition to measured fever. In contrast, the WHO ARI case definition was highly sensitive but had low specificity even after restricting the definition to require 2 rather than 1 ARI symptom. In general, case definitions based on a single respiratory symptom, which may be simpler to operationalize than multisymptom definitions, performed with moderate to high sensitivity and varying specificity. All the case definitions evaluated in this analysis had low to moderate PPVs under the relatively low prevalence conditions of the study years, underscoring the importance of influenza virus testing for accurate case identification when disease prevalence is low.

The clinical manifestation of influenza among our study participants varied in several ways when compared with a study in nonpregnant adults enrolling and testing using a broad influenza case definition, similar to PRIME. Myalgias, shortness of breath/difficulty breathing, sore throat, and nasal congestion were more prevalent in our study of pregnant women compared with symptom reports in a study of nonpregnant adults with laboratory-confirmed influenza in Singapore [32]. Fever was much less frequently reported in our study of pregnant women and report of cough was consistently, frequently reported in both studies. Although potential differences in clinical manifestation are noted, the relative performance of case definitions and the symptom predictors of rRT-PCR–confirmed influenza virus infection in our analysis are consistent with the few studies that have evaluated influenza case definitions in adults who were enrolled and tested using broad case definitions and indicate that unique case definitions for influenza detection in pregnant women are unnecessary [25, 32].

Influenza-like illness case definitions are used in a variety of settings including epidemiologic and/or virologic surveillance, studies to estimate disease incidence or describe disease epidemiology, and intervention studies. The relative importance of case-definition sensitivity versus specificity varies by its intended use [33, 34]. Our findings confirm that the WHO ILI case definition is well suited for surveillance focused on identifying circulating viruses and monitoring trends in virus circulation but is suboptimal for quantifying disease incidence since it will miss a sizeable proportion of influenza cases [30]. Expanding the WHO ILI case definition to include subjective fever to make it easier to operationalize in places with frequent antipyretic use and/or infrequent thermometer use results in only a modest reduction in specificity and increase in sensitivity. In contrast, new or worsened cough as a case definition provides high sensitivity with moderate specificity while being relatively easy to operationalize. Such attributes may be optimal for most nonsurveillance purposes such as estimation of disease burden or achievement of adequate power to evaluate disease interventions while limiting specimen collection and testing resources.

A primary strength of this study is the ability to test case-definition performance among a large, diverse, multiyear sample of pregnant women. There are no published studies comparing case definitions for influenza among pregnant women to date. Unlike some prior evaluations of influenza case-definition performance that required fever for study enrollment, this study used broader symptom criteria for respiratory specimen collection and testing, which provided a unique opportunity to look at symptoms individually and in various combinations with one another. The study also used molecular assays to optimize detection of rRT-PCR–confirmed influenza.

A limitation of this study is that it was conducted across 2 influenza seasons in which the prevalence of influenza in the study population was relatively low although consistent with prevalence among unvaccinated women in several recent influenza vaccine trials [8–10]. Because NPV and PPV vary by disease prevalence, our estimates of these measures may not be generalizable to settings with higher influenza prevalence. Additionally, the diversity of settings in which this study was conducted introduces the possibility of differences in the syntax and interpretation of certain symptoms that may vary across cultural contexts such as “chills” or “myalgias.”

Purposeful case definitions are crucial to surveillance programs and research platforms to leverage resources effectively and purposefully and to increase consistency and comparability between settings. In this study, influenza case definitions performed similarly among pregnant women in 3 countries compared with prior studies among nonpregnant adults and children, supporting the applicability of case definitions for influenza surveillance in the general population to pregnant women. As in other populations, case definitions that require measured or even subjective fever will miss a substantial proportion of influenza cases among pregnant women and may be suboptimal for studies that aim to quantify influenza disease burden or describe the full spectrum of influenza disease among pregnant women.

## Supplementary Material

Supplemental Table 1

Supplemental Table 2

## Figures and Tables

**Table 1.** Characteristics of Pregnant Women With 1 or More Respiratory Illness Episode: PRIME Cohort Study, 2017–2018

	Overall (N = 3965)		Influenza Positive (n = 309)		
	n	(Col %)	n	(Row %)	Relative Risk
Study year					
2017 (year 1)	1641	(41.4)	141	(8.6)	Ref
2018 (year 2)	2324	(58.6)	168	(7.2)	0.84
Site					
India	1585	(40.0)	115	(7.3)	Ref
Peru	1874	(47.3)	109	(5.8)	0.80
Thailand	506	(12.8)	85	(16.8)	2.32
Age, mean (SD), years	27.3 (5.7)		28.2 (6.0)		
Education^a^					
No formal education	39	(1.0)	3	(7.7)	Ref
Primary	526	(13.3)	48	(9.1)	1.19
Secondary	2060	(52.0)	138	(6.7)	0.87
Postsecondary/university	1336	(33.7)	119	(8.9)	1.16
≥1 underlying medical condition^b^	667	(16.8)	49	(7.3)	0.93
Respiratory conditions	96	(2.4)	3	(3.1)	0.40
Current smoker	141	(3.6)	9	(6.4)	0.81
Vaccinated against influenza^c^	1000	(25.2)	49	(4.9)	0.56
Parity, mean (SD)^a^	1.1 (1.2)		1.2 (1.2)		
GA at enrollment, mean (SD), weeks	17.6 (6.1)		17.5 (6.1)		
Number of ILS episodes with respiratory specimen collection					
1	2883	(72.7)	202	(7.0)	Ref
2	793	(20.0)	68	(8.6)	1.22
3	209	(5.3)	26	(12.4)	1.78
>3	80	(2.0)	13	(16.3)	2.32
Days from symptom onset to respiratory specimen collection, mean (SD)^d^	2.6 (1.7)		2.2 (1.5)		

N = 3965 pregnant women.

Abbreviations: Col, column; GA, gestational age; ILS, influenza-like symptom; PRIME, Pregnancy and Influenza Multinational Epidemiologic; Ref, reference.

a Five missing responses.

b During the prior 24 months.

c Vaccinated against influenza at least 14 days prior to report of symptom onset as verified by vaccination or medical records.

d Unit of analysis is illness episodes.

**Table 2.** Symptom Predictors of Influenza Virus Infections Among Pregnant Women With Respiratory Illness: PRIME Cohort Study, 2017–2018

	Influenza Positive (n = 310)		Influenza Negative (n = 5134)		*P*	Final Model		
	n	%	n	%		aOR^a^	95% CI	*P*
Subjective fever	85	(27)	369	(7)	<.0001*	…	…	…
Measured fever ≥38.0°C	55	(18)	131	(3)	<.0001*	4.6	3.3, 6.8	<.0001*
Chills	50	(16)	353	(7)	<.0001*	1.6	1.1, 2.3	.01*
Myalgias	206	(66)	2029	(40)	<.0001*	2.9	2.2, 3.9	<.0001*
Cough	268	(86)	3264	(64)	<.0001*	2.7	1.9, 3.9	<.0001*
Runny nose	281	(91)	4588	(89)	.48	…	…	…
Difficulty breathing	123	(40)	1567	(31)	.0007*	…	…	…
Sore throat	262	(85)	3912	(76)	.0008*	…	…	…

N = 5444 illness episodes.

* Significant at ɑ = .05.

Abbreviations: aOR, adjusted odds ratio; CI, confidence interval; PRIME, Pregnancy and Influenza Multinational Epidemiologic.

a All models include the following covariates: country, continuous age in years, influenza vaccination status for the current season defined as verified receipt of influenza vaccine at least 14 days prior to report of symptom onset, categorical education, dichotomous underlying chronic condition, time between symptom onset and swab collection, and interaction terms for country and education and country and vaccination.

**Table 3.** Performance of Individual Symptoms and Symptom Combinations for Detection of rRT-PCR–Confirmed Influenza Virus Infection, Positive-Predictive Value, and Negative-Predictive Value: PRIME Cohort Study, 2017–2018

	PPV	95% CI	NPV	95% CI
Individual constitutional symptoms				
Subjective fever	19	(15, 22)	95	(95, 96)
Measured fever ≥38.0°C	30	(23, 36)	95	(95, 96)
Chills	12	(9, 16)	95	(94, 95)
Myalgias	9	(8, 10)	97	(96, 97)
Respiratory symptoms				
Cough	8	(7, 8)	98	(97, 98)
Runny nose	6	(5, 6)	95	(93, 97)
Difficulty breathing	7	(6, 9)	95	(94, 96)
Sore throat	6	(6, 7)	96	(95, 97)
Symptom combinations				
Current WHO ILI case definition	35	(27, 43)	95	(95, 96)
Measured fever ≥38°C and cough				
Modified WHO ILI case definition 1	25	(20, 30)	95	(95, 96)
Measured fever ≥38°C or subjective fever and cough				
Modified WHO ILI case definition 2	17	(14, 20)	96	(95, 96)
Measured fever ≥38°C or subjective fever or chills and cough				
WHO ARI case definition	6	(5, 6)	100	(100, 100)
At least 1 of: cough, sore throat, runny nose, difficulty breathing				
≥2 WHO ARI case definition symptoms	6	(6, 7)	98	(97, 99)
At least 2 of: cough, sore throat, runny nose, difficulty breathing				
Final model	7	(6, 8)	99	(98, 99)
Measured fever ≥38°C or chills or cough or myalgias				

N = 5444. Abbreviations: ARI, acute respiratory infection; CI, confidence interval; ILI, influenza-like illness; NPV, negative-predictive value; PPV, positive-predictive value; PRIME, Pregnancy and Influenza Multinational Epidemiologic; rRT-PCR, real-time reverse transcription–polymerase chain reaction; WHO, World Health Organization.
