# Supplementary material for: Performance of Symptom-Based Case Definitions to Identify Influenza Virus Infection Among Pregnant Women in Middle-Income Countries: Findings From the Pregnancy and Influenza Multinational Epidemiologic (PRIME) Study
Source: Clin Infect Dis. Author manuscript; Available in PMC 2023 Oct 10. (PMC10563868; doi:10.1093/cid/ciaa1697)
Supplement: Supplemental Table 1 [file NIHMS1931252-supplement-Supplemental_Table_1.docx]

|  | Overall | | | |  | India | | | |  | Peru | | | |  | Thailand | | | |
| --- | --- | --- | --- | --- | --- | --- | --- | --- | --- | --- | --- | --- | --- | --- | --- | --- | --- | --- | --- |
|  | n=310 | | | |  | n=116 | | | |  | n=109 | | | |  | n=85 | | | |
|  | n | % | | |  | n | % | | |  | n | % | | |  | n | % | | |
| Influenza A |  |  |  |  |  |  |  |  |  |  |  |  |  |  |  |  |  |  |  |
| A(H1N1pdm)09 | 166 | ( | 54 | ) |  | 61 | ( | 53 | ) |  | 72 | ( | 66 | ) |  | 33 | ( | 39 | ) |
| A(H3N2) | 96 | ( | 31 | ) |  | 46 | ( | 40 | ) |  | 19 | ( | 17 | ) |  | 31 | ( | 36 | ) |
| A/unsubtypable | 8 | ( | 3 | ) |  | 7 | ( | 6 | ) |  | 1 | ( | 1 | ) |  | 0 | ( | 0 | ) |
|  |  |  |  |  |  |  |  |  |  |  |  |  |  |  |  |  |  |  |  |
| Influenza B |  |  |  |  |  |  |  |  |  |  |  |  |  |  |  |  |  |  |  |
| B/Victoria | 7 | ( | 2 | ) |  | 0 | ( | 0 | ) |  | 6 | ( | 6 | ) |  | 1 | ( | 1 | ) |
| B/Yamagata | 30 | ( | 10 | ) |  | 1 | ( | 1 | ) |  | 9 | ( | 8 | ) |  | 20 | ( | 24 | ) |
| B/Not lineage typed | 3 | ( | 1 | ) |  | 1 | ( | 1 | ) |  | 2 | ( | 2 | ) |  | 0 | ( | 0 | ) |
|  |  |  |  |  |  |  |  |  |  |  |  |  |  |  |  |  |  |  |  |

Supplemental Table 1. rRT-PCR Confirmed Influenza Infections by Influenza Type and Sub-Type, PRIME Cohort Study, 2017-2018, N=310 rRT-PCR Confirmed Illness Episodes
