# Supplementary material for: Performance of Symptom-Based Case Definitions to Identify Influenza Virus Infection Among Pregnant Women in Middle-Income Countries: Findings From the Pregnancy and Influenza Multinational Epidemiologic (PRIME) Study
Source: Clin Infect Dis. Author manuscript; Available in PMC 2023 Oct 10. (PMC10563868; doi:10.1093/cid/ciaa1697)
Supplement: Supplemental Table 2 [file NIHMS1931252-supplement-Supplemental_Table_2.docx]

Supplemental Table 2. Frequency of Symptom Predictors of Influenza Virus Infections Among Pregnant Women with Respiratory Illness in India, Peru and Thailand, PRIME Cohort Study, 2017-2018, N=5,444 Illness Episodes

|  | **India** | | | | | | | | |  | **Peru** | | | | | | | | | | | | |  | | **Thailand** | | | | | | | | | | | | | | | | |
| --- | --- | --- | --- | --- | --- | --- | --- | --- | --- | --- | --- | --- | --- | --- | --- | --- | --- | --- | --- | --- | --- | --- | --- | --- | --- | --- | --- | --- | --- | --- | --- | --- | --- | --- | --- | --- | --- | --- | --- | --- | --- | --- |
|  | Influenza positive | | | | Influenza negative | | | |  |  | Influenza positive | | | | | Influenza negative | | | | | |  | |  | | Influenza positive | | | | | | | | Influenza negative | | | | | |  | |  |
|  | n=116 | | | | n=1798 | | | |  |  | n=109 | | | | | n=2838 | | | | | |  | |  | | n=85 | | | | | | | | n=498 | | | | | |  | |  |
|  | n | % | | | n | % | | | p-value |  | n | % | | | | n | | % | | | | p-value | |  | | n | |  | | % | |  | | n | | % | | | | p-value | |  |
| Subjective fever | 24 | ( | 21 | ) | 187 | ( | 10 | ) | **0.0006** |  | 34 | ( | 31 | ) | 118 | | ( | | 4 | ) | **<0.0001** | |  | | 27 | | ( | | 32 | | ) | | 64 | | ( | | 13 | ) | **<0.0001** | |  |  |
| Measured fever ≥38.0 C | 7 | ( | 6 | ) | 26 | ( | 1 | ) | **0.003** |  | 27 | ( | 25 | ) | 81 | | ( | | 3 | ) | **<0.0001** | |  | | 21 | | ( | | 25 | | ) | | 24 | | ( | | 5 | ) | **<0.0001** | |  |  |
| Chills | 16 | ( | 14 | ) | 114 | ( | 6 | ) | **0.0002** |  | 29 | ( | 27 | ) | 230 | | ( | | 8 | ) | **<0.0001** | |  | | 5 | | ( | | 6 | | ) | | 9 | | ( | | 2 | ) | **0.04** | |  |  |
| Myalgias | 100 | ( | 86 | ) | 1116 | ( | 62 | ) | **<0.0001** |  | 68 | ( | 62 | ) | 836 | | ( | | 29 | ) | **<0.0001** | |  | | 38 | | ( | | 45 | | ) | | 77 | | ( | | 15 | ) | **<0.0001** | |  |  |
| Cough | 113 | ( | 97 | ) | 1562 | ( | 87 | ) | **0.0009** |  | 82 | ( | 75 | ) | 1361 | | ( | | 48 | ) | **<0.0001** | |  | | 73 | | ( | | 86 | | ) | | 341 | | ( | | 68 | ) | **0.001** | |  |  |
| Runny nose | 114 | ( | 98 | ) | 1729 | ( | 96 | ) | 0.24 |  | 97 | ( | 89 | ) | 2450 | | ( | | 86 | ) | 0.43 | |  | | 70 | | ( | | 82 | | ) | | 409 | | ( | | 82 | ) | 0.96 | |  |  |
| Difficulty breathing | 79 | ( | 68 | ) | 1006 | ( | 56 | ) | **0.01** |  | 28 | ( | 26 | ) | 485 | | ( | | 17 | ) | **0.02** | |  | | 16 | | ( | | 19 | | ) | | 76 | | ( | | 15 | ) | 0.41 | |  |  |
| Sore throat | 108 | ( | 93 | ) | 1468 | ( | 82 | ) | **0.002** |  | 88 | ( | 81 | ) | 2112 | | ( | | 74 | ) | 0.14 | |  | | 66 | | ( | | 78 | | ) | | 332 | | ( | | 67 | ) | **0.04** | |  |  |
| C: Celsius  Bold values indicate statistical significance at alpha=0.05 | | | | | | | | | | | | | | | | | | | | | | | | | | | | | | | | | | | | | | | | |  |  |
